# A note on protein expression changes in chicken breast muscle in response to time in transit before slaughtering

**DOI:** 10.1186/1477-5956-11-34

**Published:** 2013-07-24

**Authors:** Enrico Zanetti, Antonio Masi, Micaela Pivato, Serena Tolin, Anna Rita Trentin, Cem Guler, Servet Yalçin, Martino Cassandro

**Affiliations:** 1 Department of Agronomy, Food, Natural Resources, Animals and the Environment, University of Padova, Viale dell’Università 16, Padova, Legnaro 37020, Italy; 2 Padova University Proteomics Center, Via G. Orus 2b, Padova 35129, Italy; 3 Animal Science Department, Ege University, Izmir TR-35100, Turkey

**Keywords:** Chicken, Proteome, Protein expression, Pre-slaughter management

## Abstract

Aims of the research were to devise a proteome map of the chicken *Pectoralis superficialis* muscle, as resolved by two-dimensional gel electrophoresis, and to characterize protein expression changes in the soluble protein fraction in commercial conditions due to age and to time in transit before slaughtering. Broilers were reared under commercial conditions until they reached a mean 1.8 kg and 36 d, or 2.6 kg and 46 d of age. Transport to the slaughterhouse took 90 or 220 minutes. Transport-induced stress was assessed from blood metabolites and leukocyte cell counts, revealing significant changes in albumin, glucose and triglyceride concentrations, in heterophils and leukocyte counts for chickens in transit for longer, and in glucose depending mainly on age. The sarcoplasmic protein fractions were extracted from a total of 39 breast muscle samples, collected 15 min *post mortem*, for analysis by two-dimensional electrophoresis.

Image and statistical analyses enabled us to study the qualitative and quantitative differences between the samples. Twelve up- or down-regulated protein spots were detected (P < 0.05): 8 related to the age effect, 2 to time in transit, and 2 to the interaction between the two. Age and time in transit influenced the avian proteome regulating the biological processes linked to the cellular housekeeping functions, related mainly to metabolism, cell division and control of apoptosis. Principal component analysis clustering was used to assess differences between birds. Age difference discriminated between the chickens analyzed better than time in transit, which seemed to have less general impact on the proteome fraction considered here.

Isolating and identifying the proteins whose expression changes in response to transport duration and age shed some light on the biological mechanisms underlying growth and stress-related metabolism in chickens. Our results, combined with a further characterization of the chicken proteome associated with commercial chicken slaughtering management, will hopefully inspire alternative strategies and policies, and action to reduce the impact of stress related to time in transit.

## Background

Rapidly-growing chickens bred for meat have been intensively selected for over 60 years, aiming for high growth and feed conversion rates, and low slaughtering age. This selection has led to the generation of inbred strains with an accelerated growth, and a particularly enhanced development of the *pectoralis* muscles [1,2].

With the increasing demand for chicken meat, it is important for this production to comply with requirements to ensure the birds’ physical welfare, both when they are growing and when they are transferred for slaughtering. Many disorders and end-product defects have been associated with the birds’ accelerated muscle growth and inappropriate pre-slaughter management, including ascites, pulmonary hypertension, dehydration, PSE meat [3-5]. The distance they are carried from the farm to the slaughterhouse has been correlated with end-product quality parameters [6], showing a negative impact of transport-induced stress on meat pH and color.

The transportation process is considered the most stressful environmental challenge experienced by broilers [7], its magnitude depending mainly on ambient temperature and time in transit (or distance) [8-10]. Many authors have reported changes in blood metabolites associated with stress in response to time in transit. Plasma corticosterone, glucose and heterophils, and leukocyte counts are considered reliable stress indicators in chickens [11-15]. On the other hand, there are few reports in the literature on the effects of transportation on muscle tissue protein expression. Among them, Hazard et al. [16] used proteomic, transcriptomic, and metabolomic approaches to assess the molecular basis for muscle response to stress in chickens, reporting expression changes in 45 protein spots, found related mainly to the cytoskeletal structure or carbohydrate metabolism networks.

The breast muscle of chickens consists almost exclusively of fast twitch fibers and is thus an ideal tissue system for assessing protein expression dynamics, particularly those associated with growth. Protein accumulation reflects the balance between two opposing processes of protein synthesis and degradation [3]. Changes due to stress can also alter protein content in muscle, reflecting many of the biochemical processes that the organism uses to cope with changing environments or external stimuli [17], such as the stress related to the transportation and handling of broiler chickens. Protein expression in chicken breast muscle was previously studied by Zanetti et al. [18,19] with a view to characterizing different local chicken populations, but few proteome analyses on performance and stress factors in broiler chickens have been published in the scientific literature.

The aim of this study was to ascertain the protein complement of chicken skeletal muscle, as resolved by two-dimensional gel electrophoresis, in order to study protein expression changes in response to time in transit before slaughtering.

## Results and discussion

Thirty-nine samples were collected from broilers reared under commercial conditions until they reached a mean 1.8 kg and 36 d of age (young group), or 2.6 kg and 46 d (old group). The time it took to transfer them to the slaughterhouse was either 90 or 220 minutes.

For commercial meat chickens, the time in transit commonly ranges from 1 to 6 h, but it is recommended that loading, travelling and unloading take less than 2 h, and it should never exceed 4 h [20]. For control purposes, we chose a group of chickens transported within the recommended time (1.5 h), which meets the need to simulate real-life production conditions.

Blood metabolite analysis and leukocyte counts for these same broilers have already been reported by Yalçın and Güler [6], who analyzed three groups of broilers that had been transported for different periods of time, sampling the groups spending the shortest and longest time in transit (90 and 220 minutes) for proteomic analysis. Their results showed that after 220 minutes in transit the birds had higher blood albumin (1.41 vs. 1.32 g/dl), glucose (205 vs. 195 mg/dl) and triglyceride levels than after 90 minutes; when younger and older birds were compared, the effect was only significant for glucose (204 vs. 198 mg/dl), p < 0.05. This situation led to a significant Age by Transit time interaction. The highest albumin and glucose concentrations were recorded for both 36 day- and 46 day-old broilers after a longer time in transit. Blood triglycerides increased significantly with time in transit (44.39 vs. 39.10 mg/dl). The interaction between slaughtered weight and distance transported was also significant for blood triglycerides. Broilers carried a short distance had similar blood triglyceride concentrations, regardless of slaughter age (38.44 and 37.44 mg/dl for 36d and 46d birds, respectively), whereas longer transit times resulted in higher blood triglyceride concentrations for both age groups (47.44 and 45.65 mg/dl, respectively).

There was no effect of slaughter age on leukocyte counts, with the exception that younger broilers had lower basophil counts than older birds (3.49 vs. 4.09%, p < 0.05). Longer times in transit increased heterophils (25.38 vs. 21.82%) and reduced lymphocytes (65.47 vs. 68.51), but had no effect on monocytes, basophils or eosinophils.

Higher albumin concentrations in chickens spending more time in transit may be due dehydration, which reduces blood volume. Triglycerides increase in the blood of birds in transit for longer due to lipolysis in fat deposits mainly as a result of starvation and the energy demand to cope with the stressful transportation conditions. The heterophil/lymphocyte ratio is a recognized measure of stress in broilers [15,21]. In our case, longer transit times increased heterophils (H) and reduced lymphocytes (L), leading to a significant rise in H/L ratios.

On the whole, the results obtained indicated a greater tendency for stress responses in broilers in transit for longer and confirmed that time in transit influences the broilers’ metabolism, serving as a good basis for analyzing changes induced in muscle cell protein expression.

### Differential protein expression

**Figure 1:**
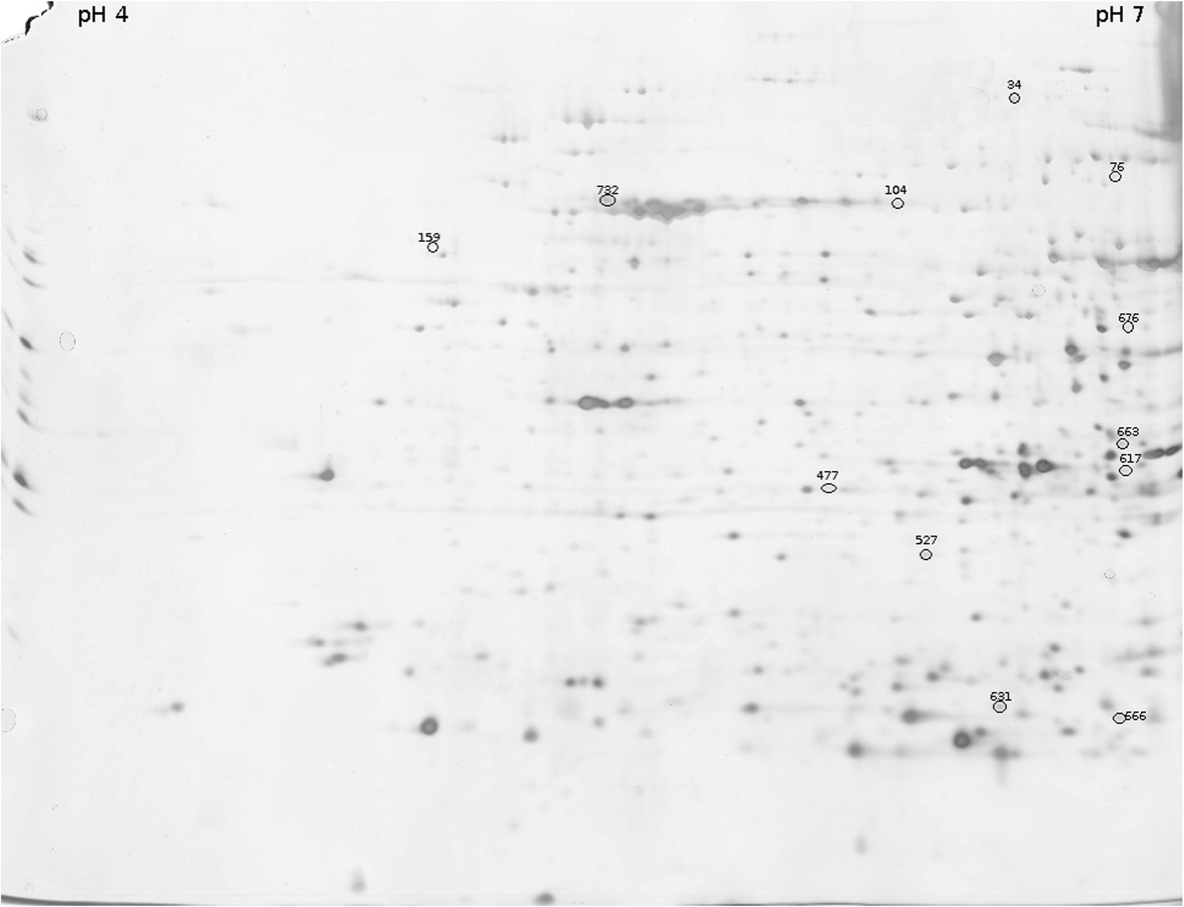
**Two-dimensional polyacrylamide gel electrophoresis of the soluble fraction of chicken breast muscle proteins.** Spot ID of down- and up-regulated proteins are shown above the spot.

Figure 1 shows the 2DE polyacrylamide gel electrophoresis findings in the *Pectoralis superficialis* muscle protein extract obtained from chickens for this analysis. To characterize the changes related to different ages and different times in transit before slaughtering, the 2DE was performed using individual samples instead of pooled group samples. This strategy was used to measure and adjust the results for individual differences because protein expression patterns can vary enormously. Here we investigated only the protein fraction that is soluble in a low ionic strength buffer in order to resolve the soluble proteins (mostly enzymes and regulatory proteins) from the structural proteins of the contractile apparatus (particularly the very abundant actin and myosin).

Using image analysis we compared 20 younger vs. 19 older birds, and 20 broilers in transit for a shorter vs. 19 in transit for longer. As shown in Figure 1, the proteins are widely distributed throughout the gel, with a slight predominance on the alkaline side. Prior to 2DE separation of all the samples, tests were conducted to assess which pH interval was the most suitable for the fraction under consideration, and since almost all of the proteome was distributed between pH 4 and 7, these conditions were chosen for the subsequent analyses, thus enabling a better separation.

Image and statistical analyses revealed that both individual and handling-induced variations were visible and measurable, but their magnitude was not as high as expected. Among the approximately 600 spots (635 for the master gel in the figure) detectable in the single gels, only 10 were differently expressed to a significant degree in the groups of samples divided by age and transit time, with a P < 0.05. Fold change ratios were selected for the purpose of protein identification when higher than the preset 1.5 cutoff, in absolute terms. No qualitative differences in protein expression (i.e. presence or absence of particular spots) were identified between the groups of chickens.

Using the fold change cutoff of 1.5, there were 35 differentially expressed spots of relevance for the age effect, and for 8 of them the difference was statistically significantly, P < 0.05 (Table 1): 7 were overexpressed in the older birds, 1 in the younger group. Supplementary information on the MS/MS identification of the relevant spots is reported in (Additional file 1: Table S1).

**Table 1:** List of up- or down-regulated proteins identified by LC-MS/MS due to the effect of age (A), time in transit (T), the interaction between age and time in transit (AxT), their function, the mean percentage volumes (%V), and the fold change ratios (FC), considering older vs. younger chickens (O/Y), longer vs. shorter transit times (L/S), and older birds in transit for longer (OL) vs. older birds with shorter transit times (OS)

**ID**^**a**^	**Effect**	**%Vol**	**P**	**FC**	**Identified protein**	**Name**	**Score**	**N. Pep.**	**Th. Mr/pI**	**Function**	**UniProt accession number**
34	A		0.045	**O/Y**-2.61	PDC6I_MOUSE	Programmed cell death 6-interacting protein	138	5	96520/6.16	Apoptosis, Cell cycle, Cell division, Protein transport, Transport	Q9WU78
104	A		0.017	3.29	ANXA6_CHICK	Annexin A6	462	13	75628/5.42	Calcium ion binding, calcium-dependent phospholipid binding	P51901
159	A		0.027	2.00	2AAA_HUMAN	Serine/threonine-protein phosphatase 2A 65 kDa regulatory subunit A alpha isoform	57	2	66065/5.00	Chromosome partition	P30153
527	A		0.022	1.61	ANXA5_CHICK	Annexin A5	352	10	36290/5.60	Negative regulation of coagulation, Calcium ion binding	P17153
617	A		0.027	1.76	PGK_CHICK	Phosphoglycerate kinase	115	4	45087/8.31	ATP binding. Glycolysis	P51903
631	A		0.021	1.87	PRDX6_CHICK	Peroxiredoxin-6	113	4	25075/5.73	Peroxidase activity. Lipid degradation	Q5ZJF4
663	A		0.029	1.85	ENOB_CHICK	Beta-enolase	72	2	47566/7.27	Phosphopyruvate hydratase activity. Glycolysis	P07322
732	A		0.016	1.58	HSP7C_CHICK	Heat shock cognate 71 kDa protein	361	10	71424/-	Stress response. ATP binding, Chaperone	O73885
77	T		0.076	**L/S** 2.08	THOP1_BOVIN	Thimet oligopeptidase	66	2	79002/5.83	Hydrolase, Metalloprotease, Protease	Q1JPJ8
477	T		0.041	1.74	G3P_CHICK	Glyceraldehyde-3-phosphate dehydrogenase	75	2	35909/8.71	Glycolysis, Apoptosis, NAD or NADH binding	P00356
671	T		0.090	1.60	HSPB1_CHICK	Heat shock protein beta-1	647	7	21715/5.77	Actin capping. Stress response	Q00649
676	T		0.041	1.61	KPYK_CHICK	Pyruvate kinase muscle isozyme	146	3	58434/7.28	ATP, magnesium and potassium binding, Pyruvate kinase activity. Glycolysis	P00548
76	AxT		0.052	**OL/OS** 2.58	PYGM_MOUSE	Glycogen phosphorylase, muscle form	226	7	97681/6.65	Carbohydrate metabolism, glycogen metabolism	Q9WUB3
666	AxT		0.094	2.04	TPIS_CHICK	Triosephosphate isomerase	290	8	26832/6.79	Fatty acid biosynthesis, Gluconeogenesis, Glycolysis, Lipid synthesis, Pentose shunt	P00940

Number of peptides identified (N.Pep.). For FC, a negative sign was added when the protein was up-regulated in the Y or S groups.

**Figure 2:**
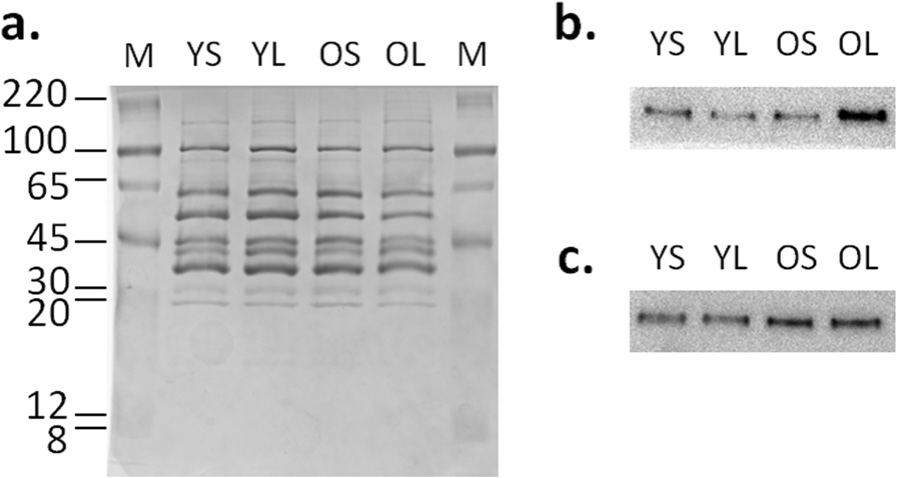
**Western blot analysis. a**. Sypro Ruby total protein staining of nitrocellulose membrane. MW of the marker (M) are given in kDa. The pooled samples from different individuals in each group was loaded, i.e. younger (Y) and older (O) broilers, and shorter (S) and longer (L) transit times. **b**. Blot stained with anti-human PYGM. **c**. Blot stained with anti-human enolase.

The older birds showed an increase in the expression of five proteins involved in energy metabolism (Table 1): phosphoglycerate kinase and beta-enolase are implicated in carbohydrate synthesis and degradation, while peroxiredoxin-6 is involved in lipid degradation. Thus, as expected, age influences the metabolic and catabolic processes occurring in the muscle cells to sustain the growth and differentiation of the muscle mass. These data were validated by Western blot analysis (Figure 2b).

Annexin A5 and A6, two proteins involved in calcium-dependent phospholipid binding, were more expressed in the older birds. They are involved in endocytosis, exocytosis and membrane scaffolding processes, and play a part in protecting membrane phospholipids from coagulation mechanisms [22,23]. The differential expression of such proteins could be considered an indicator of changes in lipid metabolism.

A protein (HSP7C), which serves as a molecular chaperone that stabilizes and prevents protein aggregation until the unfolded molecule has folded properly, is involved in the mechanics of stress response. It is found over-expressed in older broilers (fold change 1.58), probably because this group needs to cope with more stress due to environmental factors as they are growing. This protein is also reportedly involved in cellular thermotolerance induced by exposure to high temperatures or other stresses [24].

The programmed cell death 6-interacting protein (PDC6I) was found overexpressed in young birds. Studies on mouse cells have shown that this protein’s up-regulation can block apoptosis either by regulating the function of other proteins directly involved in apoptosis, or by binding to endophilins that regulate membrane shape during endocytosis [25]. Up-regulation of this protein and endophilins results in cytoplasmic vacuolization, which may be partly responsible for protecting against cell death. This finding suggests that younger birds would be better protected against stress and cellular apoptosis than older ones. Lastly, older chickens exhibited a higher expression of serine/threonine-protein phosphatase 2A (2AAA), a protein required for proper chromosome segregation and for centromeric localization of shugoshin-like 1 protein in mitosis, presumably indicating an augmented cell division and muscle development pattern [26].

Ten differently expressed spots were detected that related to the effect of time in transit. After statistical analysis, the differences were only significant for two spots, P < 0.05 (Table 1). Broilers in transit for longer had a higher expression of some proteins related to energy metabolism - such as pyruvate kinase muscle isozyme (KPYK), and glyceraldehyde-3-phosphate dehydrogenase (G3P) – indicating that longer times in transit impose a higher energy demand on to the chicken to cope with harsh management conditions, hunger, temperature differences, shaking, noise and social disruption.

Another investigation on the chicken muscle proteome had already reported that KPYK was differently-expressed in chicken muscles retaining a high water holding capacity, and slow vs. fast growth, together with triosephosphate isomerase (TPIS) and housekeeping proteins like the heat shock proteins (HSP) [27]. The reported results confirm that these proteins are involved in the metabolic mechanisms that chickens use to cope with environmental changes of various kinds. With a 0.05 < P < 0.10, two more proteins join the scene, i.e. thimet oligopeptidase (THOP1) and heat shock protein B1 (HSPB1); the expression of the latter, HSPB1, which is involved in stress responses, in the breast muscle cells was also stimulated by the longer time in transit.

The Age x Transit time (AxT) interaction revealed no significant differences (P < 0.05) in proteome component expression. Using a threshold of 0.05 < P < 0.10, however, the analysis indicated that older broilers in transit for longer had a higher content of two proteins involved in the energy metabolism pathway (glycogen phosphorylase and triosephosphate isomerase), while younger broilers showed no differences in relation to time in transit.

### Western blot validation

To validate the differences in protein expression identified by our proteomic investigations, Western blot analyses were conducted on samples from broilers belonging to the different groups considered. Total protein normalization was preferred, rather than housekeeping protein normalization, for several reasons. This was primarily because of the limited availability of suitable antibodies, which are usually produced against human or murine antigens and their cross-reactivity to avian species would have to be tested (e.g. there was no positive feedback when the antibody against the human housekeeping protein histone H2B was tested). In addition, beta-tubulin (one of the most often used housekeeping proteins) could not be used as a control because it only occurs as a contaminant in the sarcoplasmic protein fractions due to its limited solubility in low ionic strength saline buffers [28]. Numerous articles have reported on the effectiveness of total protein normalization as an alternative to choosing specific housekeeping proteins [29-31].

Several polyclonal antibodies against differently expressed proteins were tested for their cross-reactivity with avian antigens. Among them, anti-PYGM and anti-enolase (produced against the homolog human proteins) recognized the avian protein. Proteomic analysis showed a difference in the expression of these two proteins between the groups of birds of different weights (Figure 1 and Table 1). The Western blots of PYGM and enolase (Figures 2b and 2c, respectively) are shown together with the densitometric analysis (Table 2) of the blots and total protein staining (Figure 2a). The data are consistent with the variations seen in 2D gels: for PYGM (spot 76, Figure 1) in particular, the fold change (OL/OS) in protein abundance calculated using 2D gel analysis was 2.58 (Table 1), which is fairly consistent with the fold change of 1.94 obtained from the Western blot densitometric analysis (Table 2). Similarly, enolase (spot 663, Figure 1) shows a fold change (O/Y) of 1.85 (Table 1), which seems amply consistent with the fold changes of 2.4 (OL/YL) and 1.68 (OS/YS) calculated by Western blot densitometry.

**Table 2:** **Volume intensity, measured as pixels *per *area considered, were calculated using the image lab 3.0 (Biorad) software, drawing boxes of equal area around the whole lanes of the Sypro Ruby stained membrane, or close to the blot bands**

	**Volume intensity**			**Blot/SyproR%**	
	**Sypro ruby**	**PGYM**	**Enolase**	**PGYM**	**Enolase**
**YS**	94,874,384	3,791,340	767,025	4.00	0.81
**YL**	107,562,330	3,738,332	679,206	3.48	0.63
**OS**	84,668,383	5,148,930	1,150,182	6.08	1.36
**OL**	79,410,020	9,381,180	1,200,996	11.81	1.51

Values refer to Figure 2a for total protein quantification, and to Figures 2b and 2c for visualization with anti-human PYGM and anti-human enolase antibodies, respectively. The ratios of the blot band to total protein densitometric values are reported.

### Principal component analysis

**Figure 3:**
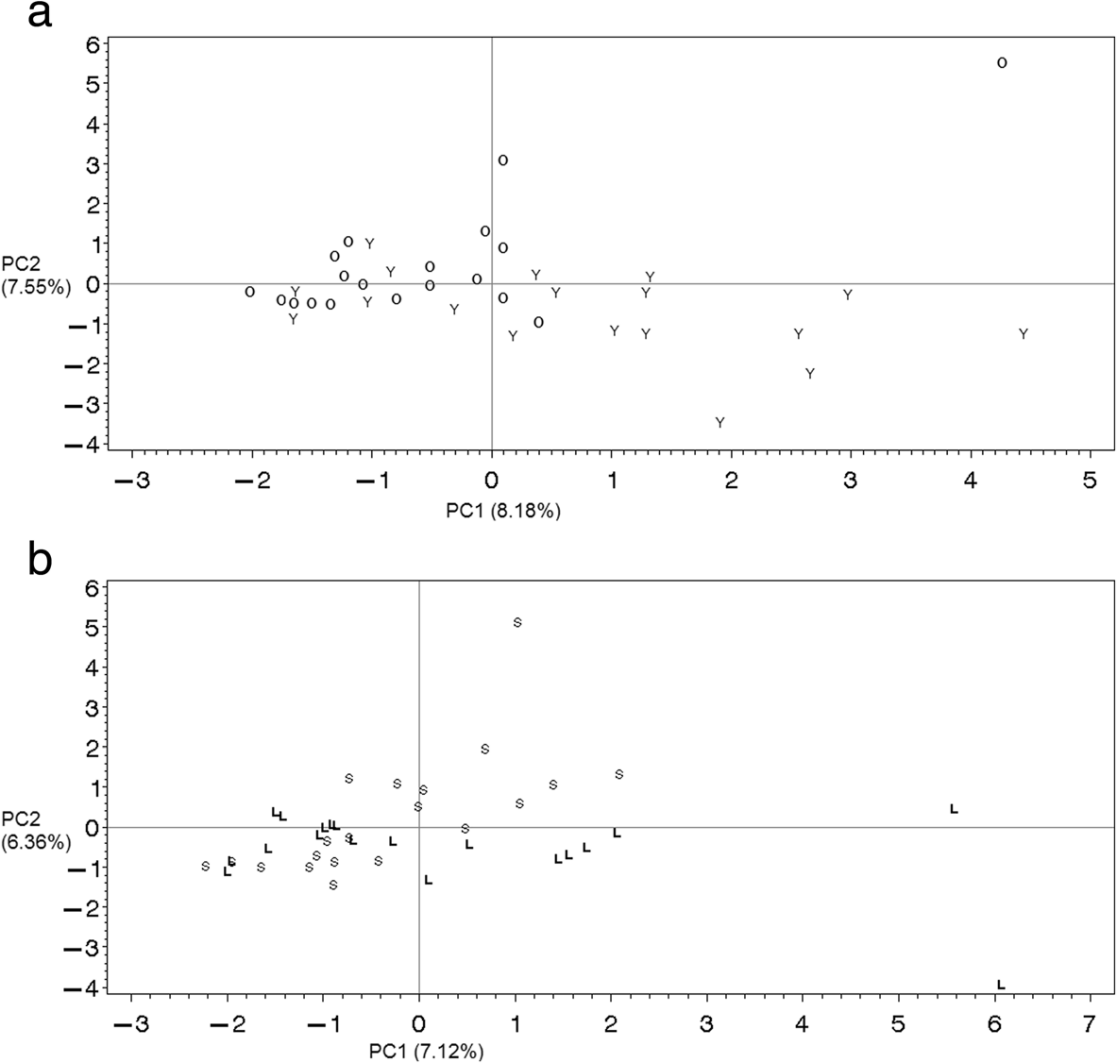
**Principal component analysis scores based on the differently expressed spot volumes. a**. 2D scores plot for older and younger (Y) broilers. **b**. Scores plot for broilers experiencing shorter (S) and longer (L) transit times.

Principal component analysis was used to study individual groupings based on the similarity of their protein expression. Figures 3a and 3b show the two-dimensional plot of the first and second principal component scores for the old vs. young birds, and for the birds with a shorter vs. a longer transit time, respectively. The first principal component explained 8.18% and 7.12% of the total variation for Age and Transit time, respectively, the second principal component explained 7.55% and 6.36%. All proteins detected with 2-DE were included in the analysis. In theory, birds sharing similar patterns of protein expression can be considered more similar than birds showing greater differences in protein expression (and indirectly in their genes). Our approach aimed to cluster together birds sharing the same characteristics, e.g. body weight and time in transit in the present case. When only statistically significant spots were considered, there was no clear clustering (data not shown), meaning that all proteins - even those whose expression differed slightly – could provide little useful information for the purposes of our analysis.

The age difference seemed to discriminate between the chickens analyzed more than the time in transit, which generally appeared to have less impact on the proteome fraction considered here. In any case, the effects studied do not seem to be strong enough to stimulate a differential protein expression, as was the case in a previous study on breed differentiation, which showed a more distinct variation [18].

## Conclusion

Our findings clearly indicate that broilers in transit for a lengthy period of time had to cope with higher stress levels, as demonstrated by their blood parameters and changes in their protein expression profile, which were statistically significant although they were restricted to a limited number of proteins. The changes observed point to metabolic adjustments in the birds to provide energy and thus cope in the short term with temperature changes and food deprivation.

Isolating and identifying the proteins involved in these processes shed some light on the underlying biological mechanisms, although the variations observed were not as evident as in similar studies focusing on the muscle proteome [32].

Current commercial production strategies do not enable critical steps in meat chicken management to be handled in an optimal manner. Global strategies being adopted focus on reducing and mitigating the steps with a heavy impact on animal welfare and product quality. That is why we judged it to be more realistic to compare the changes occurring in the birds after two different transit times. From a proteomic standpoint, our chickens proved not particularly susceptible to a difference in transit time of 130 minutes so, with a view to maximizing their different responses, it would be useful to test broader time intervals (although this would mean departing from the real-life working conditions of commercial slaughterhouses). Tissues other than those chosen for this study (e.g. liver, brain and adrenal glands) might also be more responsive and therefore more suitable for studying stress-related proteomic changes.

Our results support the conviction that appropriate animal handling, in accordance with national and EU regulations, can prevent stress in the birds. Blood metabolite levels confirmed that these domestic birds can be screened for stressful treatment and are reliable indicators of susceptibility to stress, but only proteome analysis can reveal the metabolic pathways involved and the complex networks of interaction.

On the other hand, age had a stronger influence on the avian muscle proteome and the variations that we observed suggest that adjustments occur in the regulation of biological processes relating to the cellular housekeeping functions, linked mainly to metabolism, cell division and the control of apoptosis.

Many proteins involved in responses to age and stress presumably remain to be identified, but this study can help pave the way to future, larger investigations on the chicken skeletal muscle proteome with a view to filling the gap in our understanding between the identification of the most relevant proteins expressed and the explanation for the biological differences.

Together with a further characterization of the chicken proteome associated with stressful conditions suffered by commercial chickens, the results presented here will hopefully inspire alternative strategies, policies and actions to improve animal welfare when it comes to their pre-slaughter management. This topic will be of greater importance in the near future, as the number of authorized poultry slaughterhouses is declining all over in Europe, and the mean time in transit for the birds is consequently increasing – and so is the consumer’s concern for animal welfare.

## Methods

### Broilers

The study was conducted in the spring on Ross 308 broiler stock slaughtered at one of the commercial processing plants in Turkey. The animal care practices used in the experiments were in accordance with the principles of the Ege University Animal Research Ethics Committee and national law (no. 5199). The broiler chickens were reared under commercial conditions at the same farm until they weighed 1800 ± 50 g and were 36 days old (young group), or they weighed 2600 ± 50 g and were 46 days old (old group). The chickens were reared under a lighting regime of 23 h of light and 1 h of dark, and feed and water were provided for consumption *ad libitum* with standard broiler rearing, growing and finisher diets. All broilers were sampled from the same integrated poultry company from October to November. Food was withdrawn 12 h before transportation to the slaughterhouse. Birds were marked by leg tags and collected in cages and carried in two different trucks to the commercial slaughterhouse. Cage piling was designed so as to prevent scalding of the chickens by using low stacking densities and allowing sufficient air circulation between the cages. The outside temperature was between 15° and 18°C with a relative humidity of 62%. The transit time was 90 (short) or 220 minutes (long). The shorter transit time was considered as the baseline condition for transportation to the slaughterhouse. The broilers were allowed to rest for 1 h before being slaughtered in identical conditions, i.e. they were electrically stunned, bled, scalded, defeathered, and eviscerated. Ten carcasses from each group, balanced for gender, were randomly sampled.

### Sample collection and protein extraction

Five grams of breast muscle (*Pectoralis superficialis*) were collected from the left half of 10 carcasses from each group 15 min after death and frozen in liquid nitrogen for analysis. The sarcoplasmic protein fraction was extracted using a procedure modified from Rathgeber et al. [33]. One-gram samples of breast meat, pulverized in liquid nitrogen, were homogenized in 20 mL of LIS buffer (0.05 M potassium phosphate, 1 mM NaN3, 2 mM EDTA, pH 7.3, 2°C) for 10 s, and placed on ice for 30 min. These samples were centrifuged at 17,500 g for 15 min at 2°C. Ten mL of supernatant containing the sarcoplasmic proteins were removed 2 cm from the bottom of the tube. The remaining supernatant was discarded and the pellet was resuspended in an additional 20 mL of LIS buffer, homogenized and centrifuged as described previously. The protein content of the sarcoplasmic protein extract was determined using the bicinchoninic acid method (Sigma-Aldrich, GmbH, Munich, Germany).

### Two-dimensional electrophoresis

Two-dimensional electrophoresis was completed on a total of 78 samples (2 replicates per animal). Protease inhibitors (80-6501-23, GE Healthcare, Uppsala, Sweden) were added to the LIS protein extract in an Amicon Ultra 4 Millipore and centrifuged at 7,500 g for 15 min at 3°C. Two mL of UHQ water containing protease inhibitors were added to the concentrate and the centrifugation step was repeated. Isoelectric focusing was done using an Ettan IPGphor II with a Manyfold tray (GE Healthcare). In a final volume of 450 μL, 300 μg of protein were loaded onto immobilized pH gradient strips (Immobiline DryStrip pH 4-7, 24 cm, GE Healthcare). Proteins were loaded by including an adequate volume of extract in a buffer consisting of 7 M urea, 2 M thiourea, 2% (w/v) CHAPS, 0.2% (w/v) DTT and 0.2% carrier ampholytes (Sigma-Aldrich). Strips were loaded by overnight passive rehydration. For the subsequent IEF, the voltage was increased gradually to 10,000 V until a total of 80,000 Vh was reached. Prior to SDS–PAGE, strips were equilibrated for 15 min in a reducing solution containing 2% DTT, 6 M urea, 30% glycerol, 2% SDS and 50 mM Tris-Cl pH 8.8, followed by a 15 min step in an alkylation solution comprising 5% (w/v) iodoacetamide, 6 M urea, 30% (v/v) glycerol, 2% (w/v) SDS, 50 mM Tris-Cl pH 8.8, and bromophenol blue as a dye (Sigma-Aldrich). SDS–PAGE was performed in an Ettan DALT*six* electrophoresis unit (GE Healthcare) on 12% polyacrylamide gels (2.6% C) at 40 mA/gel, until the dye track reached the bottom of the gels. Proteins were fixed for at least 1 h in 10% methanol and 7% acetic acid. The fixing solution was changed at least twice to wash the gel. Gels were silver stained using the Shevchenko et al*.* protocol [34].

### Image analysis

Gel images were acquired with a densitometric ImageScanner II (Amersham Biosciences, UK) and analyzed using computerized image analysis software, Image Master 2D Platinum (GE Healthcare, Uppsala, Sweden). On each gel, automatic spot detection was then validated by manual spot editing. After the matching process, individual expression profiles were obtained, averaging the replicates for each bird, and percentage volumes of the spots were recorded for each individual to study individual differences. Detected and matched spots were normalized by expressing the relative quantity of each spot as the ratio of the individual spot quantity to the total quantity of valid spots. The mean of the two values obtained by running the tests in duplicate was calculated for each sample and each spot.

### Statistical analysis

The following threshold criteria were applied to select only good-quality protein spots for expression profiling: each protein spot had to be present in both the biological samples tested, and detected in at least 60% of the gels in a given group (higher or lower weight birds, and longer or shorter times in transit). Only spots with more than 1.5-fold changes in volume after normalization within each group (by weight and transit time) were considered as altered and further validated with the nonparametric Wilcoxon-Mann–Whitney test using the NPAR1WAY of SAS® [35]. Spot expression was considered significant if P < 0.05.

Principal component analysis was performed using PROC PRINCOMP of SAS, including all expressed spots.

### In-gel digestion, protein identification and database search

The spots of interest were excised from the gels, dehydrated with acetonitrile for 10 min and then dried in SpeedVac. Cysteines were reduced with 10 mM dithiothreitol in 50 mM NH4HCO3 for 1 h at 56°C, and alkylated with 55 mM iodoacetamide for 45 min at room temperature in the dark. Gel pieces were repeatedly washed with 50 mM NH4HCO3 and acetonitrile and then dried under a vacuum. Proteins were digested in gel using sequencing grade modified trypsin (Promega, Madison, WI). 10 μL of trypsin (12.5 ng/μL in 50 mM NH4HCO3) were added to each spot, and the samples were incubated for 30 min at 4°C. Digestion was continued at 37°C overnight. The peptides obtained after trypsin digestion were extracted for 3 times with 50 μL of 50% acetonitrile/1% formic acid, dried under a vacuum and dissolved in 10 μL of 0.1% formic acid. Liquid chromatography-tandem mass spectrometry (LC-MS/MS) analyses were performed with a 6520 Q-TOF mass spectrometer (Agilent Technologies, Santa Clara, CA, USA) coupled to a chip-based chromatographic interface. Four μL of the samples were injected into the chip and loaded into the enrichment column (C18, 4 mm, 40 nL volume) at a flow rate of 4 μL/min. Peptides were separated in the C18 nano-column (43 mm × 75 μm) at a flow rate of 0.5 μL/min. Water/formic acid 0.1% and acetonitrile/formic acid 0.1% were used as eluents A and B, respectively. The chromatographic separation was done using a gradient of eluent B from 3% to 50% in 15 min. Mass spectra were acquired in a data-dependent mode: MS/MS spectra of the 3 most intense ions were acquired for each MS scan in the range of 350-2400 m/z. Scan speed was set to 4 MS spectra/sec and 3 MS/MS spectra/sec. Capillary voltage was set to 1750 V and drying gas to 5 l/sec. Raw data files were converted into Mascot Generic Format (MGF) files with MassHunter Qualitative Analysis Software version B.03.01 (Agilent Technologies) and searched using the Mascot Search Engine (version 2.2.4 Matrix Science, London, UK) against the SwissProt database (version 2010_09, 519348 sequences, 183273162 residues). Enzyme specificity was set to trypsin with one missed cleavage using a mass tolerance window of 10 ppm for the precursor ion and 0.05 Da for the fragment ions. Carbamidomethylcysteine and oxidation of methionine were selected as the fixed and variable modification, respectively. Proteins with at least 2 peptides and significant individual scores (p < 0.05) were considered as having been positively identified.

### Western blot analysis

Mono-dimensional gel electrophoresis (1-D Tris-Gly SDS-PAGE) was done with home-made 11% T polyacrylamide mini-gels. Samples were prepared as described above, and equal amounts of protein from different bids in the same group were pooled together to normalize individual variability. Fifty μg of each pool were loaded in the system. After SDS-PAGE runs, gels were trans-blotted onto nitrocellulose membrane (GE Healthcare Life Sciences Whatman, Germany) using a semi-dry system. As described by Aldridge and colleagues [29], nitrocellulose membranes were stained with Sypro Ruby Stain (Invitrogen) following the manufacturer’s recommendations, and the image was captured prior to cutting the membrane at different MW for visualization with different antibodies. After saturation in 5% milk in 10 mM Tris, 0.14 mM NaCl, 0.05% Tween 20, pH 7.4 for 2 hours, the membranes were incubated with anti-human PYGM and anti-human enolase antibodies (Santa Cruz Biotechnology, Santa Cruz, CA) for two hours at 1:100 and 1:200 dilution, respectively. Goat anti-rabbit IgG coupled with horseradish peroxidase (Santa Cruz Biotechnology) was used as a secondary antibody (with 90 min of incubation), and the Western Blotting Luminol Reagent (Santa Cruz Biotechnology, cat. sc-2048) was used for detection purposes. To ensure an equal sample loading in each lane, protein content was verified using the bicinchoninic assay method prior to SDS-PAGE, and further assessed by means of densitometric analyses on the gel bands after Sypro Ruby staining, according to Aldridge and colleagues [29].

Gel images were captured and analyzed using the ChemiDoc XRS + imager with Image Lab™ 3.0 software (Biorad). The integrated optical density value (volume intensity), defined as the Σ of each pixel value, was ascertained for equal-sized boxes drawn around bands. Boxes around the whole lane were considered for the total protein analysis, while boxes of the same size close to the band were used for quantification using Western blot.

## Competing interests

The authors have no competing interests to declare.

## Authors’ contributions

EZ performed the proteomic analysis and contributed to its design, the 2-DE and gel image analysis, and the statistical analysis on the proteomic data, and drafted the manuscript. AM contributed to the overall experimental design of the proteomic analysis and to the preparation of the manuscript. ST and MP performed the LC-MS/MS analyses and identified the relevant spots; MP and ART ran the Western blot analyses. SY and CG conceived the overall experimental design, and managed the chicken growth and sampling phases. MC generally coordinated the study, contributed to the proteomic experiment design, and was involved in fund raising and manuscript preparation. All authors have read and approved the final manuscript.

## Supplementary Material

Additional file 1: Table S1 Supplementary information on the MS/MS identification of the relevant spots: number of peptides identified (N.Pep), Mascot score (Score), charge state (Ch.), mass over charge of the ions observed (Ion obs), calculated and theoretical peptide mass (Pep mass calc, pep mass th), number of missed cleavages (Miss. Cleav), and peptide sequences. Click here for file
