# Supplementary material for: A note on protein expression changes in chicken breast muscle in response to time in transit before slaughtering
Source: Proteome Sci. 2013 Jul 24;11:34. doi: 10.1186/1477-5956-11-34 (PMC3728074; doi:10.1186/1477-5956-11-34)
Supplement: Additional file 1: Table S1 — Supplementary information on the MS/MS identification of the relevant spots: number of peptides identified (N.Pep), Mascot score (Score), charge state (Ch.), mass over charge of the ions observed (Ion obs), calculated and theoretical peptide mass (Pep mass calc, pep mass th), number of missed cleavages (Miss. Cleav), and peptide sequences. [file 1477-5956-11-34-S1.docx]

**Additional file 1: Table S1.** **Supplementary information on the MS/MS identification of the relevant spots: number of peptides identified (N.Pep), Mascot score (Score), charge state (Ch.), mass over charge of the ions observed (Ion obs), calculated and theoretical peptide mass (Pep mass calc, pep mass th), number of missed cleavages (Miss. Cleav), and peptide sequences**

| **ID** | **Identified protein** | **N.Pep** | **Score** | **Ion obs** | **Pep mass calc** | **Ch.** | **Pep mass th** | **Δ ppm** | **Miss. cleav** | **Pep score** | **Exp value** | **Sequence** |
| --- | --- | --- | --- | --- | --- | --- | --- | --- | --- | --- | --- | --- |
| 34 | PDC6I_MOUSE | 5 | 138 | 450.2297 | 898.4448 | 2 | 898.4436 | 0.0012 | 0 | 25.03 | 0.16 | FTDLFEK |
|  |  |  |  | 465.7522 | 929.4898 | 2 | 929.493 | -0.0033 | 0 | 29.14 | 0.26 | DLQQSIAR |
|  |  |  |  | 481.2676 | 960.5206 | 2 | 960.524 | -0.0034 | 0 | 30.37 | 0.15 | DTVLSALSR |
|  |  |  |  | 484.2364 | 966.4582 | 2 | 966.4593 | -0.0011 | 0 | 21.37 | 0.54 | CSDIVFAR |
|  |  |  |  | 532.8033 | 1063.5921 | 2 | 1063.5913 | 0.0008 | 0 | 26.86 | 0.22 | LALASLGYEK |
| 76 | PYGM_MOUSE | 7 | 226 | 369.7043 | 737.394 | 2 | 737.3959 | -0.002 | 0 | 24.08 | 0.37 | VSELYK |
|  |  |  |  | 373.6859 | 745.3572 | 2 | 745.3606 | -0.0034 | 0 | 29.5 | 0.15 | VEDVER |
|  |  |  |  | 411.7249 | 821.4352 | 2 | 821.4395 | -0.0044 | 0 | 31.59 | 0.16 | TIAQYAR |
|  |  |  |  | 458.7459 | 915.4773 | 2 | 915.4774 | 0 | 0 | 37.23 | 0.028 | NLAENISR |
|  |  |  |  | 459.737 | 917.4595 | 2 | 917.4607 | -0.0011 | 0 | 21.57 | 1.1 | APNDFNLK |
|  |  |  |  | 527.2893 | 1052.5641 | 2 | 1052.5655 | -0.0014 | 0 | 26.02 | 0.23 | VIFLENYR |
|  |  |  |  | 531.7776 | 1061.5406 | 2 | 1061.5427 | -0.0021 | 0 | 44.61 | 0.005 | MSLVEEGAVK |
|  |  |  |  | 539.7738 | 1077.5331 | 2 | 1077.5376 | -0.0045 | 0 | 23.58 | 0.54 | M(ox)SLVEEGAVK |
| 104 | ANXA6_CHICK | 13 | 462 | 394.229 | 786.4434 | 2 | 786.4487 | -0.0053 | 0 | 23.92 | 0.79 | DLIADLK |
|  |  |  |  | 445.7564 | 889.4982 | 2 | 889.5022 | -0.004 | 0 | 33.15 | 0.08 | DAFVAIVR |
|  |  |  |  | 448.7465 | 895.4784 | 2 | 895.4804 | -0.002 | 0 | 21.1 | 0.64 | VFQEFVK |
|  |  |  |  | 494.2684 | 986.5222 | 2 | 986.5284 | -0.0062 | 0 | 22.59 | 1.1 | DLLEAGELK |
|  |  |  |  | 498.2376 | 994.4607 | 2 | 994.4607 | -0.0001 | 0 | 23.32 | 0.39 | AELSGDFEK |
|  |  |  |  | 502.7387 | 1003.4629 | 2 | 1003.4644 | -0.0015 | 0 | 40.36 | 0.0061 | ALLALCGGDD |
|  |  |  |  | 505.2782 | 1008.5419 | 2 | 1008.5426 | -0.0007 | 0 | 34.14 | 0.045 | FLSILCTR |
|  |  |  |  | 536.8036 | 1071.5926 | 2 | 1071.5924 | 0.0002 | 0 | 26.87 | 0.32 | SEIDLLNIR |
|  |  |  |  | 537.2518 | 1072.489 | 2 | 1072.4825 | 0.0065 | 0 | 38.14 | 0.0066 | STAEYFAER |
|  |  |  |  | 545.2717 | 1088.5288 | 2 | 1088.535 | -0.0062 | 0 | 28.24 | 0.2 | DAIAGIGTDEK |
|  |  |  |  | 546.2744 | 1090.5343 | 2 | 1090.5328 | 0.0015 | 0 | 23.96 | 0.71 | SEIDMLDIR |
|  |  |  |  | 550.337 | 1098.6595 | 2 | 1098.6583 | 0.0012 | 0 | 49.87 | 0.0008 | MLVVLLQGAR |
|  |  |  |  | 910.4053 | 1818.7961 | 2 | 1818.8007 | -0.0046 | 0 | 22.25 | 0.18 | EEDDVVSEDLVEQDAK |
| 159 | 2AAA_HUMAN | 2 | 57 | 437.2225 | 872.4305 | 2 | 872.4352 | -0.0047 | 0 | 22.96 | 0.53 | NEDVQLR |
|  |  |  |  | 580.7935 | 1159.5724 | 2 | 1159.5721 | 0.0003 | 0 | 34.34 | 0.049 | LTQDQDVDVK |
| 527 | ANXA5_CHICK | 10 | 352 | 373.1919 | 744.3692 | 2 | 744.3766 | -0.0074 | 0 | 32.65 | 0.098 | ADAEALR |
|  |  |  |  | 378.2716 | 754.5286 | 2 | 754.5316 | -0.003 | 0 | 22.73 | 0.012 | LLLAVVK |
|  |  |  |  | 424.7342 | 847.4538 | 2 | 847.4552 | -0.0014 | 0 | 42.79 | 0.012 | DAQVLFR |
|  |  |  |  | 447.2386 | 892.4627 | 2 | 892.4654 | -0.0027 | 0 | 23.7 | 0.74 | QEIASAFK |
|  |  |  |  | 451.7433 | 901.4721 | 2 | 901.4756 | -0.0036 | 0 | 29.42 | 0.2 | VDEALVEK |
|  |  |  |  | 500.2763 | 998.538 | 2 | 998.5396 | -0.0016 | 0 | 29.21 | 0.12 | TPAEVQNIK |
|  |  |  |  | 501.3013 | 1000.588 | 2 | 1000.5917 | -0.0037 | 0 | 28.3 | 0.17 | VLTEILASR |
|  |  |  |  | 537.2925 | 1072.5705 | 2 | 1072.5764 | -0.0059 | 0 | 28.39 | 0.32 | SEIDLLDIR |
|  |  |  |  | 567.2733 | 1132.5321 | 2 | 1132.536 | -0.0039 | 0 | 40.39 | 0.0093 | GAGTDDDTLIR |
|  |  |  |  | 588.2825 | 1174.5505 | 2 | 1174.554 | -0.0035 | 0 | 36.88 | 0.017 | ALLLLCGGDDE |
| 617 | PGK_CHICK | 4 | 115 | 366.2171 | 730.4196 | 2 | 730.4225 | -0.003 | 0 | 32.55 | 0.15 | VVEVTGK |
|  |  |  |  | 369.2026 | 736.3907 | 2 | 736.3942 | -0.0034 | 0 | 12.35 | 10 | AAGFLMK |
|  |  |  |  | 403.232 | 804.4494 | 2 | 804.4494 | 0 | 0 | 31.72 | 0.11 | FVEVVGR |
|  |  |  |  | 528.7995 | 1055.5844 | 2 | 1055.5863 | -0.0019 | 0 | 38.7 | 0.013 | VLPGVDALSSV |
| 631 | PRDX6_CHICK | 4 | 113 | 453.7365 | 905.4584 | 2 | 905.4607 | -0.0023 | 0 | 12.39 | 13 | NFDEILR |
|  |  |  |  | 507.3045 | 1012.5945 | 2 | 1012.5957 | -0.0012 | 0 | 20.44 | 1.1 | LPFPIIADK |
|  |  |  |  | 511.2892 | 1020.5639 | 2 | 1020.5644 | -0.0006 | 0 | 25.93 | 0.37 | VVFIFGPDK |
|  |  |  |  | 596.3373 | 1190.66 | 2 | 1190.6659 | -0.0059 | 0 | 26.96 | 0.25 | LSILYPATTGR |
| 663 | ENOB_CHICK | 2 | 72 | 437.7291 | 873.4436 | 2 | 873.4443 | -0.0007 | 0 | 27.39 | 0.23 | IEEALGDK |
|  |  |  |  | 554.7852 | 1107.5559 | 2 | 1107.556 | -0.0001 | 0 | 44.68 | 0.0046 | AAIAQAGYTDK |
| 732 | HSP7C_CHICK | 10 | 361 | 383.209 | 764.4035 | 2 | 764.4068 | -0.0034 | 0 | 31.65 | 0.11 | VQVEYK |
|  |  |  |  | 387.2086 | 772.4026 | 2 | 772.4079 | -0.0053 | 0 | 25.4 | 0.62 | DNNLLGK |
|  |  |  |  | 387.7199 | 773.4252 | 2 | 773.4283 | -0.0031 | 0 | 41.53 | 0.021 | NTTIPTK |
|  |  |  |  | 402.724 | 803.4334 | 2 | 803.4389 | -0.0054 | 0 | 22.54 | 1.4 | ITITNDK |
|  |  |  |  | 417.7029 | 833.3913 | 2 | 833.3953 | -0.0039 | 0 | 23.58 | 0.42 | MVQEAEK |
|  |  |  |  | 429.7296 | 857.4447 | 2 | 857.4494 | -0.0048 | 0 | 30.75 | 0.082 | GTLDPVEK |
|  |  |  |  | 472.7649 | 943.5152 | 2 | 943.5161 | -0.0009 | 0 | 29.55 | 0.29 | VCNPIITK |
|  |  |  |  | 497.2651 | 992.5156 | 2 | 992.5178 | -0.0022 | 0 | 35.29 | 0.037 | EIAEAYLGK |
|  |  |  |  | 600.34 | 1198.6655 | 2 | 1198.667 | -0.0015 | 0 | 59.94 | 0.00015 | DAGTIAGLNVLR |
|  |  |  |  | 614.8155 | 1227.6165 | 2 | 1227.6207 | -0.0043 | 0 | 52.61 | 0.00059 | VEIIANDQGNR |
| 477 | G3P_CHICK | 2 | 75 | 371.707 | 741.3995 | 2 | 741.4021 | -0.0025 | 0 | 30.19 | 0.063 | AAADGPLK |
|  |  |  |  | 685.3776 | 1368.7406 | 2 | 1368.7361 | 0.0045 | 0 | 44.99 | 0.0036 | GAAQNIIPASTGAAK |
| 666 | TPIS_CHICK | 8 | 290 | 365.7067 | 729.3987 | 2 | 729.4021 | -0.0033 | 0 | 26.48 | 0.51 | AIADNVK |
|  |  |  |  | 374.1969 | 746.3792 | 2 | 746.381 | -0.0018 | 0 | 30.6 | 0.22 | EAGITEK |
|  |  |  |  | 425.735 | 849.4555 | 2 | 849.4596 | -0.0041 | 0 | 27 | 0.33 | VVFEQTK |
|  |  |  |  | 562.2812 | 1122.5478 | 2 | 1122.5492 | -0.0013 | 0 | 51.36 | 0.00086 | IGVAAQNCYK |
|  |  |  |  | 419.8759 | 1256.606 | 3 | 1256.6109 | -0.005 | 0 | 19.38 | 0.97 | SHVSDAVAQSTR |
|  |  |  |  | 663.3266 | 1324.6386 | 2 | 1324.6445 | -0.0059 | 0 | 27.7 | 0.2 | IIYGGSVTGGNCK |
|  |  |  |  | 486.9135 | 1457.7186 | 3 | 1457.7151 | 0.0036 | 0 | 39.22 | 0.014 | HVFGESDELIGQK |
|  |  |  |  | 489.5772 | 1465.7098 | 3 | 1465.7161 | -0.0063 | 0 | 28.04 | 0.19 | TATPQQAQEVHEK |
| 676 | KPYK_CHICK | 3 | 146 | 495.7559 | 989.4972 | 2 | 989.5029 | -0.0058 | 0 | 38.03 | 0.023 | GSGTAEVELK |
|  |  |  |  | 615.3203 | 1228.626 | 2 | 1228.6299 | -0.0039 | 0 | 50.35 | 0.0012 | LDIDSEPTIAR |
|  |  |  |  | 680.3564 | 1358.6982 | 2 | 1358.6976 | 0.0005 | 0 | 57.26 | 0.00031 | NTGIICTIGPASR |
| 77 | THOP1_BOVIN | 2 | 66 | 404.6985 | 807.3825 | 2 | 807.3875 | -0.005 | 0 | 20.12 | 1.2 | QDVYQR |
|  |  |  |  | 567.3041 | 1132.5936 | 2 | 1132.5989 | -0.0052 | 0 | 39.85 | 0.016 | QANTGLFNLR |
| 671 | HSPB1_CHICK | 7 | 647 | 920.42 | 919.42 | 2 | 919.42 | 23.6 | 0 | 53 | 0.0019 | DWYHGSR |
|  |  |  |  | 1088.54 | 1087.53 | 2 | 1087.53 | 22.7 | 0 | 72 | 3.8e-005 | QDEHGFISR |
|  |  |  |  | 1373.77 | 1372.77 | 2 | 1372.77 | 21.8 | 0 | 98 | 7.8e-008 | YTLPPGVEATAVR |
|  |  |  |  | 1410.69 | 1409.68 | 2 | 1409.68 | 20.3 | 0 | 70 | 4.6e-005 | WPSGSAWPGYFR |
|  |  |  |  | 1501.87 | 1500.86 | 2 | 1500.86 | 21.7 | 1 | 97 | 5.7e-008 | KYTLPPGVEATAVR |
|  |  |  |  | 1810.01 | 1809.01 | 2 | 1809.01 | 21.7 | 0 | 111 | 2.2e-009 | VTLDVNHFAPEELVVK |
|  |  |  |  | 1825.02 | 1824.01 | 2 | 1824.01 | 20.2 | 0 | 147 | 6.3e-013 | LLPSESALLPAPGSPYGR |
